# Potential Use of Wearable Inertial Sensors to Assess and Train Deep Cervical Flexors: A Feasibility Study with Real Time Synchronization of Kinematic and Pressure Data during the Craniocervical Flexion Test

**DOI:** 10.3390/s23083911

**Published:** 2023-04-12

**Authors:** Elena Bocos-Corredor, Tomás Pérez-Fernández, Raquel Perez-Dominguez, Sonia Liébana, Susan Armijo-Olivo, Rafael Raya, Aitor Martin-Pintado-Zugasti

**Affiliations:** 1 Departamento de Fisioterapia, Facultad de Medicina, CEU San Pablo University, 28668 Madrid, Spain; 2 Departamento de Tecnologías de la Información, Escuela Politécnica Superior, CEU San Pablo University, 28668 Madrid, Spain; 3 Faculty of Business and Social Sciences, University of Applied Sciences, 53604 Osnabrück, Germany

**Keywords:** assessment technology, biomechanics feasibility studies, movement disorders, neck pain

## Abstract

The aim of the study was to develop a novel real-time, computer-based synchronization system to continuously record pressure and craniocervical flexion ROM (range of motion) during the CCFT (craniocervical flexion test) in order to assess its feasibility for measuring and discriminating the values of ROM between different pressure levels. This was a descriptive, observational, cross-sectional, feasibility study. Participants performed a full-range craniocervical flexion and the CCFT. During the CCFT, a pressure sensor and a wireless inertial sensor simultaneously registered data of pressure and ROM. A web application was developed using HTML and NodeJS technologies. Forty-five participants successfully finished the study protocol (20 males, 25 females; 32 (11.48) years). ANOVAs showed large effect significant interactions between pressure levels and the percentage of full craniocervical flexion ROM when considering the 6 pressure reference levels of the CCFT (*p* < 0.001; η^2^ = 0.697), 11 pressure levels separated by 1 mmHg (*p* < 0.001; η^2^ = 0.683), and 21 pressure levels separated by 0.5 mmHg (*p* < 0.001; η^2^ = 0.671). The novel time synchronizing system seems a feasible option to provide real-time monitoring of both pressure and ROM, which could serve as reference targets to further investigate the potential use of inertial sensor technology to assess or train deep cervical flexors.

## 1. Introduction

Neck pain is a very common musculoskeletal disorder, considered as the fourth main cause of disability worldwide. Neck pain has an annual prevalence that ranges from 15% to 75.1% [1] and it has been estimated that 71% of the adults suffers at least one neck pain episode in their lifetime [2]. Neck pain is also related to older age [1] and shoulder pain [3].

Craniocervical pain has been associated with multiple alterations of the cervical sensorimotor system when compared to the asymptomatic population, such as impaired endurance [4] and strength of the neck muscles [5,6], altered proprioception [7,8] and kinematics [9,10], reduced active range of movement (ROM) [11], or changes in muscle morphology [12].

The craniocervical flexion test (CCFT) is focused on the contracting ability of longus capitis and longus colli as deep cervical flexors (DCFs) [13], which appear to provide stability and support to the cervical region that may be relevant for optimal sensorimotor control of the cervical region [14]. It consists of performing the craniocervical flexion movement while maintaining an isometric and progressive contraction through five incremental stages of pressure [13]. Correct performance of the test requires activation control and the ability to maintain the contraction during the stages without the occurrence of compensatory movements such as retraction, low cervical flexion, or overactivation of superficial cervical flexors [13].

Alterations in the structure and function of the DCF, including alterations during the performance of the CCFT, have been reported in patients with craniofacial pain, neck pain, temporomandibular disorders, and cervicogenic headache [15,16,17]. Consequently, the function of the DCFs is often considered in the assessment of patients with neck pain [18,19] and when prescribing exercise programs [20].

The construct validity of the CCFT has been investigated by EMG using nasopharyngeal electrodes, showing a progressive linear relationship between the amount of deep muscle activation and the five stages of the test [15,16,17].

The CCFT requires the clinician to analyze the motion of the head and the superficial muscle activity by palpating or observing any compensatory movements. The degree of craniocervical flexion ROM should progressively increase during the five consecutive phases of the CCFT, which represents the increasing demand of DCF muscle contraction for contraction of the DCF muscles [13,21]. One of the main signs of abnormal patterns is the lack of an increase in head rotation with progressive increments of the test, which is believed to occur because the movement strategy becomes more of a head retraction action [13,15].

The use of wearable inertial sensors has been shown to be an easy-to-use option to objectively measure and track the ROM associated with each phase of the CCFT [22]. This technology could prevent the occurrence of abnormal head retraction patterns, due to the information received by a computer screen guiding the performance of the test for both the patient and the examiner. In addition, inertial sensors associated with computer feedback could provide a potential future alternative option for assessing and retraining the DCF muscles. A recent study using wearable inertial sensor technology aimed to standardize a set of craniocervical flexion ROM targets equivalent to each of the pressure stages of the CCFT. Although reliability was proven for measuring ROM during the CCFT, the high variability in the amount of craniocervical ROM limited the possibility of standardizing a set of ROM targets for each of the stages of the CCFT. However, the instrumentation used in that study only provided data for ROM at each of six pressure stages of the CCFT and the process of associating values for ROM and pressure was not automatically synchronized by a computer [22].

It is our hypothesis that a novel computer-based real-time synchronization approach using high-precision inertial technology and pressure sensors will allow for better discrimination and standardization of ROM targets equivalent to each of the pressure levels generated by the pressure biofeedback unit used for the CCFT. Moreover, it would allow for the development of a new biofeedback of synchronized visual graphics of pressure and ROM displayed on a computer screen that could be used for research and clinical purposes when assessing or training this movement in patients with neck-related disorders. Inertial sensor technology has shown positive results in previous research investigating the assessment or management of multiple pain conditions [23,24].

The objectives of this study were: (a) to develop a novel real-time, high-precision (computer-based) time synchronization system to continuously record pressure and craniocervical flexion ROM during the CCFT and (b) to assess the feasibility of the system for measuring synchronized ROM and pressure data during the CCFT and discriminating the values of ROM between different pressure levels.

## 2. Materials and Methods

### 2.1. Design

This was a descriptive, observational, cross-sectional, feasibility study in a sample of asymptomatic participants. Participants should be asymptomatic to accomplish the objectives of the study, which is based on describing normative values of ROM and pressure during an ideal execution of the CCFT. These data could later serve as a reference for patients with several pain conditions.

This study was accepted by the Research Ethics Committee of Ceu San Pablo University (236/17/08). Participants gave their written informed consent before enrolling in the study and could withdraw at any time during the protocol, in accordance with the Declaration of Helsinki and WHO standards [25].

### 2.2. Sample and Selection

Participants were recruited via social networks, flyers, and emails within the University of San Pablo Ceu. Asymptomatic subjects between 18 and 65 years of age were included if they were able to correctly complete all the levels of the CCFT (detailed below). Therefore, subjects who were unable to perform the 3 maximal repetitions of the craniocervical flexion movement due to any compensation strategy or without reaching 30 mmHg were excluded, as were those who did not perform the all the stages of the CCFT correctly.

Subjects were interviewed and screened by a clinician to assess if they presented with any of the following exclusion criteria: (a) previous surgery in the neck or head area, (b) pain in the neck or head region and/or shoulders at the time they participated in the study or during the last year, (c) previous diagnosis of headaches and temporomandibular disorders, or (d) any neurological deficits.

If deemed eligible, participants were invited to participate and asked to sign the informed consent.

### 2.3. Instrumentation and Measures

For all measures of full-range craniocervical flexion and the CCFT described in the testing procedure (see below), subjects were positioned in a supine position with knees flexed, the forearms resting on the abdomen, and the neck in a neutral position. The assessor placed a deflated pressure pad (Pressure Biofeedback Unit, Stabilizer™ Chattanooga Group, Hixon, TN, USA) beneath the neck of the subject and inflated it to 20 mmHg. A closed loop connected the pressure biofeedback unit with a small, low-range industrial pressure sensor (IPSL-M12, © RS Components Ltd., Corby, UK). Specifically, this sensor provides an analogue output in a range of 0–5 V and has an accuracy of up to ±0.25% FS, in a working pressure range of 50 to 1000 mbar. An Arduino UNO microprocessor was used to process the analogue value of the pressure, where its approximation to mmHg was calculated to be transmitted, via a serial port, to a Bluetooth module. Finally, the pressure value was transmitted to a web application (see below) for its visualization and storage, with a frequency of 50 Hz.

In a pre-study phase, the calibration of the pressure sensor was carried out considering the temperature conditions that were going to occur during the laboratory trials. For this purpose, a high-precision digital manometer (±0.05% FS; 1/4″ NPT M, 5 PSI; DPG210, Omega Engineering Inc., Norwalk, CT, USA) was introduced into the closed loop and established as a reference for the calculation of a regression line, whose input values are volts and output values are mmHg. This same digital manometer connected to the pressure biofeedback unit has been used in previous research and was associated with good to excellent reliability, ranging from 0.78 to 0.96 depending on the stage and examiner, when used for providing a pressure reference associated with craniocervical flexion ROM values during the CCFT [22].

A 4 cm × 4 cm × 8 cm wireless wearable inertial sensor (Werium Solutions©, Madrid, Spain) weighing less than 200 g was adhered to the forehead of the participant defined as the point where the lines that bisect the forehead horizontally and longitudinally cross. This inertial sensor allowed real-time tracking of the ROM progressive increase while performing craniocervical flexion required for the CCFT, whilst it was shown on a computer screen. This measuring instrument has previously demonstrated good to excellent inter-rater and intra-rater reliability in the assessment of global neck [26] or craniocervical flexion ROM [22]. It recorded the degrees of motion of flexion from the starting neutral position of the neck cervical ROM achieved when the inertial sensor was calibrated at zero.

Pressure and inertial motion capture sensors were synchronized in terms of processing, visualization, and storage of their data. A web application was developed using HTML and NodeJS technologies. The data transmitted by both instruments were received through parallel processes, in which each value was stored, synchronously, at a frequency of 50 Hz. Therefore, the software application computed the range of motion of the participants (expressed as degree angles from the calibrating starting position) and a paired value of pressure (mmHg) sampled every 20 ms from each sensor simultaneously (inertial and pressure) during the performance of the CCFT by each patient. This information was exported in .csv using RStudio (library ‘readr’). Data from each participant included multiple records of ROM and pressure continuously registered during the whole time period spent to perform the CCFT.

The possible occurrence of neck retraction compensatory strategies during the testing procedure was prevented by using a thin pressure sensitive textile mat sensor (Pressure mat dev kit, Sensing Tex^®^, Barcelona, Spain) behind the head, which showed real-time pressure data on a separate computer screen. This mat has been used in previous research [22]. Any increase in pressure levels higher than 0.75 kg was considered a compensation in neck retraction, so participants were asked to repeat the test or excluded because of an unsuccessful CCFT performance. This cut-off value for maximal increments in weight has been described in previous research [21].

### 2.4. Testing Procedure

After filling in the informed consent, all subjects completed a form to record the demographic data regarding sex, age, height, and weight. Once completed, the assessor explained how to correctly perform a full-range CCF movement and the CCFT (detailed below).

One assessor guided and supervised the correct performance of the testing procedure and communicated with participants throughout the process. An independent examiner monitored and recorded the data from the sensors and provided technical support for their use.

Once the subjects were placed in the supine position, they were asked to maintain the neutral cervical position for the placement of the inertial sensor and the biofeedback computer screen to ensure correct visualization by the participants as well as the maintenance of the neutral position. At that time, the examiner inflated the pressure cuff to 20 mmHg as the baseline pressure level when the inertial sensor was calibrated at zero. Then, participants performed 3 full-range repetitions of the CCF movement to assess the patients’ capacity to correctly reach 30 mmHg of pressure and to record the maximal ROM achieved, which was later used as a reference point to calculate the percentage of movement relative to the maximum.

Next, the CCFT was performed based on the clinical protocol described by Jull et al. [13]. Participants were instructed to gently and slowly perform a nodding action to raise the target pressure from baseline (20 mmHg) to first stage (22 mmHg) and to hold for 3 s before relaxing and returning to the initial position. This process was repeated through each 2 mmHg increase in the pressure level of the test until reaching 30 mmHg. The subjects performed a maintained contraction at each of the stages without showing any associated compensation such as excessive activation of the superficial musculature or retraction. The examiner assessed possible compensations through observation and palpation and assisted by providing verbal or visual clues to guide the process.

The biofeedback computer screen allowed for real-time assessment of the correct performance of the 3 maximal repetitions of CCF and the CFFT by displaying pressure and ROM graphics at the same time. It also served as biofeedback for the patients, who were instructed about using the pressure graphic as a reference for the pressure levels they should reach. The examiner visually assessed the presence of any retraction compensation or excessive superficial muscle activation, and confirmed that both pressure and ROM values displayed on the tablet screen synchronously increased to reach each pressure target. Subjects who showed an incorrect execution of the CCFT were excluded from the study.

### 2.5. Data Analysis Plan

Data were analyzed with the Statistical Package for the Social Sciences (SPSS) version 27.0 (SPSS Inc., 233 S Wacker Dr, 11th Fl, Chicago, IL 60606, USA). A normal distribution of quantitative data was evaluated by the Kolmogorov–Smirnov test (*p* > 0.05). Descriptive data of the sample are expressed as mean and standard deviation (SD) for continuous variables or as number of cases and percentage for categorical variables.

First, pressure values (mmHg) were rounded to one decimal place and craniocervical flexion ROM (degrees) was calculated and expressed as a percentage relative to the full range of craniocervical flexion. Then, the recorded pressure (mmHg) and the craniocervical flexion ROM (expressed in degrees and as a percentage relative to the full ROM) were submitted to a one-way analysis of variance (ANOVA) with the 6 pressure levels of the CCFT (20 (baseline) and 22, 24, 26, 28, 30 mmHg) as the factor and the continuous data of ROM percentage as the dependent variable. Another two independent ANOVAs repeated this calculation with 11 pressure levels (from 20 to 30 mmHg separated by 1 mmHg) and 21 pressure levels (from 20 to 30 mmHg separated by 0.5 mmHg) as factors. Then, these same three ANOVAS were used including the values of ROM expressed in degrees as the dependent variable. Post hoc statistical tests (Bonferoni) were used to assess differences in ROM between consecutive pressure levels in all ANOVAs.

Statistical significance was set at *p* < 0.05 and the effect sizes were calculated as eta squared (η^2^), interpreted as thresholds for small, medium, and large effect sizes values of 0.01, 0.06, and 0.14 respectively [27]. Descriptive data of ROM at each pressure level are expressed as mean ± SD, standard error of the mean (SEM), and 95% confidence interval (CI).

Finally, a regression model with curvilinear estimation was used to evaluate the relation between pressure and ROM when both were analyzed as continuous variables with two decimal points.

### 2.6. Sample Size Calculation

The sample size of this study was determined using G*Power, V.3.1.9.2 (Franz Faul, University at Kiel, Germany), considering the results from a pilot study with 10 asymptomatic participants. Sample size was calculated using a one-way ANOVA with 0.95 power (1-beta error probability) and an alpha level of 0.5, considering the mean and SD values of the % of full craniocervical flexion ROM obtained at 6 independent pressure points (20, 22, 24, 26, 28, and 30 mmHg). A sample size of 36 individuals was estimated considering an effect size of 0.87. Considering the probability of technical errors related to the automatic recording of data from inertial or pressure sensors, an approximate additional 20% of patients was estimated (*n* = 45).

## 3. Results

A total of 63 participants accepted to participate in the study and were screened for possible eligibility. Eighteen were excluded based on the following criteria: pain in the neck/head region and/or shoulders at the time of the measurement (*n* = 4), or not being able to perform the CCFT by progressively increasing the pressure and ROM at each stage until the test was completed (*n* = 14). Finally, we included a total of 45 participants who successfully finished the study protocol (20 males, 25 females; mean [SD] age, 32 (11.48) years) (Table 1).

One-way ANOVAs showed large effect significant interactions between pressure levels and the percentage of full craniocervical flexion ROM when considering the 6 pressure reference levels of the CCFT (F = 5035.65; *p* < 0.001; η^2^ = 0.697), 11 pressure levels separated by 1 mmHg (F = 3316.66; *p* < 0.001; η^2^ = 0.683), and 21 pressure levels separated by 0.5 mmHg (F = 2836.47; *p* < 0.001; η^2^ = 0.671). Similar results were observed when considering the interaction between craniocervical flexion ROM measured in degrees and pressure levels of the CCFT (F = 4056.53; *p* < 0.001; η^2^ = 0.650), 11 pressure levels separated by 1 mmHg (F = 2806.81; *p* < 0.001; η^2^ = 0.646), and 21 pressure levels separated by 0.5 mmHg (F = 2583.97; *p* < 0.001; η^2^ = 0.650).

Table 2 shows the descriptive data corresponding to the ROM associated with pressure levels separated by 1 mmHg, both expressed as degrees of ROM and as a percentage relative to the full ROM. SDs showed great variability between subjects, while 95%CI and standard errors (SEs) were low due to the large sample of pressure and ROM time points measured. SEs of the percentage of ROM do not overlap between any of consecutive pressure levels separated by 1 mmHg.

Table 2 also shows that all post hoc differences were significant when comparing ROM percentage between consecutive levels of pressure separated by 2 mmHg (pressure stages of the CCFT) or when comparing between consecutive levels separated by 1 mmHg, except for the comparison between 27 and 28 mmHg. Post hoc analysis also showed statistically significant differences between all consecutive pressure levels separated by 0.5 until 27 mmHg. However, pressure levels above 27 mmHg did not show differences in ROM percentage between consecutive pressure levels separated by 0.5 mmHg.

**Figure 1:**
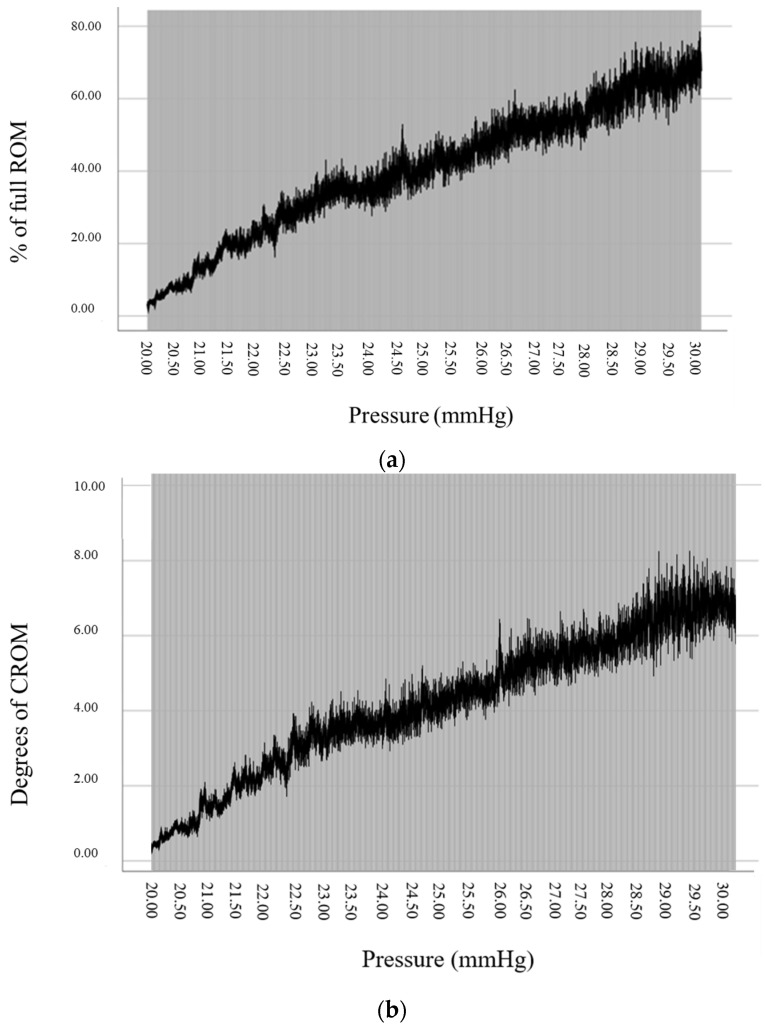
(**a**) Relationship between % of full CCROM and pressure; (**b**) relationship between degrees of CCROM and pressure. Data are represented as mean ± 2 * SE. CCROM: craniocervical flexion range of motion.

Curve estimation regression analysis showed a significant curvilinear relationship (quadratic model) between pressure and ROM, both measured in percentage (R = 0.811; *p* < 0.001) and in degrees (R = 0.805; *p* < 0.001). Figure 1a,b show the curvilinear quadratic trend of the relationship between pressure and ROM, in which the curve seems to slightly flatten at higher values of pressure.

## 4. Discussion

The present study showed the development of a novel time synchronizing system that allowed for continuous high-precision synchronous computer recording of pressure and ROM during the performance of the CCFT.

Previous research has investigated the ROM necessary to reach each separate stage of the CCFT using digital imaging techniques [16,21] or inertial sensor technology [22]. In these previous studies, ROM and pressure were assessed separately and the relationship between both variables was later analyzed by examiners. The time synchronization system developed in the present study allowed the autonomous recording of objective continuous data of both variables by a computer; furthermore, it was not limited to only six pressure levels of the CCFT and was not dependent on a posterior analysis of the association between both variables performed by researchers.

Moreover, this synchronizing system allowed the display on the same tablet screen of real-time visual feedback of both ROM and pressure graphs that could be easily observed by the participant and by examiners. This option specially facilitated the performance of the CCFT, since one of the components of the test is to visually assess the quality and ROM to confirm that it proportionally increases with progressive stages of the test [13,17]. This visual assessment is usually a challenge for clinicians, since the changes in ROM between consecutive stages are usually very small, as can be noted by looking at differences in ROM between consecutive CCFT stages shown in Table 2.

The use of similar time synchronization systems with computer feedback could be part of future research or clinical practice in order to improve the precision of the assessment or training of DCFs, and to limit the occurrence of altered movement strategies of pushing the neck and head back into the pressure sensor (retraction) during the CCFT. This retraction strategy is considered one of the main compensations during the performance of the CCFT. Previous research has observed that patients with neck pain perform less craniocervical flexion ROM to reach each pressure level of the CCFT compared to asymptomatic participants, suggesting that patients with neck pain performed additional neck retraction and supporting the clinical guideline to assess the quality and quantity of movement during the CCFT [15].

The descriptive mean values of full-range ROM percentage necessary to reach each stage of the CCFT in our study (Table 2) seem similar to those previously reported in young adults when measured with digital imaging techniques [28] or inertial sensor technology [22]. Moreover, previous research also showed that the amount of craniocervical flexion progressively increases during the five successive stages of the CCFT [15,16,17,21,22]. In contrast, other studies have observed values of full-range ROM percentage higher than those of our study, especially in the last 2 stages of pressure (28 and 30 mmHg), where their values were approximately 10% [17] or 15% [21] greater than those in our study. These differences could be explained by the fact that participants in our study had increased values of the full range of craniocervical flexion ROM (10.59°) compared to these studies (8.5°) [21], so the percentage of the full ROM necessary to reach the target pressure level was reduced. Differences between studies regarding the mean values of full-range craniocervical flexion ROM could be explained by examiner criteria on the subjective cutoff point of maximum ROM that the patient can achieve without signs of altered movement strategies, such as head retraction or excessive use of superficial flexors.

Regression analyses in our study showed a quadratic (curvilinear) relationship between pressure values and craniocervical flexion ROM. This result agrees with previous research that also found similar quadratic correlations between the craniocervical flexion ROM and the five incremental stages of the CCFT [15]. The quadratic relationship occurs because the increases in ROM seem to occur to a lower extent at higher levels of pressure, so the correlation between both variables is not linear, but a curve that progressively flattens as pressure levels increase (Figure 1). This trend of lower changes in ROM needed to progress through higher pressure stages of the CCFT may be the natural way the relationship between craniocervical flexion movement and the flattening of the cervical lordosis occurs in healthy population. If this is the case, it can be hypothesized that the last degrees of full range craniocervical flexion motion are associated with an increased flattening of lordosis when compared to the first part of the motion. However, another explanation for this phenomenon could be that the increased demand for DCFs during the last stages of the test force some patients to use compensatory strategies of retraction to increase the pressure during the test. In the case of the existing population, these compensations should be small, as they were not detected by the pressure textile mat sensor, or by the real-time monitoring of the simultaneous pressure and ROM increases observed in the computer screen feedback.

The smaller changes in ROM necessary to progress through the last stages of the test indicated that the ANOVAs showed less discriminative capacity to differentiate changes in ROM between consecutive 0.5 mmHg changes in pressures above 27 mmHg. Regardless, the data for ROM percentage or ROM in degrees allowed finding differences between consecutive changes of 1 and 2 mmHg throughout the pressure range. Despite the great variability between subjects, these results showed the high precision of the instruments used, and suggest that the values of ROM shown in Table 2 accurately describe a set of craniocervical flexion ROM targets that could be used in future research to investigate the potential use of ROM as the guide to assess or train DCFs. The use of technology of this purpose, such as inertial sensors, could provide some advantages, including the fact that its use limits the possibility of patients using compensatory strategies of retraction to progress through the stages of the test. The retraction movement may barely affect the values of craniocervical flexion ROM registered by the inertial sensors, since it mainly implies a posterior translation of the sensor, but not necessarily an angular movement on the sagittal plane, which is the motion registered by the inertial sensor.

The use of technology with reference ROM targets to assess or train DCFs could also potentially allow for its use in positions other than supine, which might provide alternative options for progression of exercises targeting DCFs using biofeedback. The CCFT is performed in the supine position because the air-filled pressure cuff needs to be compressed between the cervical lordosis and the bed, so it could be limited when used as an exercise to progress towards training in functional postures and tasks [29]. Clinical practice guidelines for neck pain management recommend the individualization and progressive prescription of exercise for patients with chronic neck pain with movement coordination impairments [30]. It is believed that better outcomes are achieved if each patient is regarded as an individual, and exercise approaches are based on identification of patient-specific tailored interventions [31].

The possible use of craniocervical flexion ROM biofeedback to guide the assessment or training of DCFs implies that the computer biofeedback information would be focused on the tilting motion of the head instead of the flattening of the lordosis, which is the basis of the biofeedback provided by the CCFT biofeedback pressure unit. The results of the present study and previous research [16,17,21,22,28] provided evidence about the relationship between lordosis flattening and the craniocervical flexion motion. Future research could further investigate how the craniocervical flexion ROM is associated with DCF electromyographic activity by using an approach similar to that of our study, including real time synchronization of both variables.

This study has several limitations. First, the results are limited to the characteristics of asymptomatic subjects included from a university community. Although this sample may be the most suitable to describe normative values in a healthy population who can correctly perform the CCFT, it is unclear how factors such as gender, age, or anatomical characteristics could explain the variability between subjects and affect the results. Previous research did not observe differences between young and elder participants regarding the amount of craniocervical flexion ROM during the CCFT [28]. Future research could also make comparisons between subjects with craniocervical pain conditions and the asymptomatic population. Therefore, due to the variability in the mentioned characteristics, this model could be insufficient to demonstrate its universality for patients with neck pain.

Second, the muscle activation of superficial muscles while performing the CCFT was assessed by examiner palpation and observation, but was not objectively monitored by surface electromyography.

## 5. Conclusions

The novel time synchronizing system developed in this study seems a feasible option for future research and clinical practice to provide real-time monitoring of both pressure and ROM through high-precision feedback shown on a computer screen during the performance of the CCFT.

The precision of the system for measuring synchronized ROM and pressure data, as well as the capacity to discriminate values of ROM between different pressure levels of the CCFT, allow for the use of the values of craniocervical flexion ROM presented in this study as reference targets to further investigate the potential use of wearable inertial sensor technology to assess or train DCFs.

Furthermore, curve estimation regression analysis showed a significant curvilinear relationship between pressure and ROM.

## Figures and Tables

**Table 1:** Demographic characteristics of the participants.

n		45
Gender (%)	Female	25 (55.5)
	Male	20 (44.4)
Age		32.0 ± 11.5
Height (m)		1.0 ± 0.1
Weight (kg)		68.7 ± 14.9

Data expressed as mean ± standard deviation or as absolute and relative values (%).

**Table 2:** Descriptive data for craniocervical ROM associated with 1 mmHg levels of pressure.

Pressure Level (mmHg)	ROM Expressed in % of Full ROM		ROM Expressed in Degrees	
	Mean ± SD [CI]	SEM	Mean ± SD [CI]	SEM
20	3.6 ± 10.0 [3.3–3.8]	0.12	0.4 ± 1.2 [0.4–0.5]	0.01
21	11.2 ± 13.3 [10.6–11.7] *	0.3	1.1 ± 1.4 [1.1–1.2] *	0.03
22	20.7 ± 13.3 [20–21.4] *^	0.36	2.2 ± 1.5 [2.1–2.3] *^	0.04
23	28.5 ± 12.5 [27.6–29.3] *	0.42	2.9 ± 1.43 [2.8–3.0] *	0.05
24	30.7 ± 12.8 [29.8–31-6] *^	0.44	3.2 ± 1.4 [3.0–3.3] *^	0.05
25	38.1 ± 14 [37.0–39.1] *	0.54	4.0 ± 1.4 [3.9–4.1] *	0.06
26	45.2 ± 15.7 [44.1–46.3] *^	0.56	4.7 ± 1.5 [4.60–4.79] *^	0.05
27	50.6 ± 16.7 [49.2–51.9] *	0.68	5.3 ± 1.9 [5.2–5.4] *	0.08
28	52.2 ± 16.4 [50.9–53.5] ^	0.67	5.6 ± 1.9 [5.5–5.8] *^	0.08
29	58.4 ± 18.1 [56.5–60.3] *	0.97	6.0 ± 1.8 [5.8–6.1] *	0.1
30	61.6 ± 16.4 [59.9–63.2] *^	0.83	6.1 ± 1.7 [6.0–6.2] ^	0.08
Full ROM	100%	-	10.6 ± 2.9 [9.8–11.5]	0.43

mmHg = millimeters of mercury; SD = standard deviation; CI = confidence interval; SEM = standard error of measurement; * Statistically significant differences with the immediately inferior pressure level (1 mmHg difference); ^ Statistically significant differences with the inferior pressure CCFT stage separated by 2 mmHg (only for 22, 24, 26, 28, and 30 mmHg).

## Data Availability

Not applicable.
